# The German Impact of Future Events Scale (IFES-S): Adaption and Validation for Clinical Samples

**DOI:** 10.3389/fpsyt.2019.00813

**Published:** 2019-11-18

**Authors:** Julia Kroener, Caroline Schaitz, Anna Maier, Bernhard Connemann, Zrinka Sosic-Vasic

**Affiliations:** Department of Psychiatry and Psychotherapy III, University Clinic of Ulm, Ulm, Germany

**Keywords:** imagery, assessment, questionnaire, prospective imagery, flash-forwards

## Abstract

Prospective intrusive mental images occur frequently among several psychological disorders. Their assessment is an important tool since the imagination of events can drive future behavior, such as suicidal acts. One valid measure evaluating those prospective images is the impact of future events scale (IFES). However, to date, there is no German equivalent to the English IFES. Therefore, the aim of the present study was to adapt and validate the IFES at hand of a clinical sample and a healthy control sample.After translation, item analyzes were conducted, and as a result, four items were excluded from further analysis resulting in the German short version of the IFES, the IFES-S. Construct validity of the German IFES-S was tested through correlational analysis with convergent and divergent measures. Specificity and sensitivity were assessed through ROC analysis. The German IFES-S showed good internal consistency for the overall measurement with a Cronbach’s α of .93. Additionally, it displayed good convergent and divergent validity. An optimal cutoff score of 23 was established to discriminate between clinical populations and healthy controls. In summary, the German IFES-S promises to be a valid self-report instrument for the assessment of prospective intrusive imagery within the context of clinical samples.

## Introduction

Strong and accumulating evidence demonstrates the association between imagery processes and mental disorders (1–4). Intrusive mental images of past experiences are widely known within the clinical context through clinical disorders such as posttraumatic stress disorder (PTSD) where individuals experience flashbacks (5); or social phobia (6), where individuals experience recurrent imageries of past aversive social experiences, which are connected to the onset of the disorder. The occurrence of intrusive mental imagery within psychiatric disorders has been frequently linked to increased burden of disease (e.g., 7). One possible mechanism behind this finding is the understanding that mental imagery are termed to evoke greater emotional responses than simple language based representations (3, 8, 9). Furthermore, Holmes & Mathews (10) showed that mental imagery was more likely to evoke affective responses within the investigated participants than was verbal processing.

However, investigations in recent years have focused on prospective intrusive imaginations, defined as “the experience of involuntary, distressing mental images of events in the future that come to mind unbidden” (11, p. 201). Interestingly, the brain does not seem to differentiate either between the imagination of past or future events, or between imaginations and actual real-life perceptions, as the same neuronal pathways are activated during both mental occurrences (12–16).

This new perspective on imagery stresses the importance of including the concept of imaginary pre-experiencing in terms of “flash-forwards” (17) or prospective intrusive imagery, rather than only focusing on re-experiencing past situations by means of flashbacks, intrusive thoughts, or memories, in order to gain a more holistic insight into the possible impact of prospective intrusive imagery within the clinical setting. Past research has supported this notion by indicating that mental imagery can drive human behavior (18–20): Participants within these studies were more likely to display and accomplish future behavior, if they were imagining the accomplishment thereof beforehand through the lens of a third-person perspective. This finding is especially important within the context of suicidal ideation, where prospective mental imagery in terms of suicidal flash-forwards seems to play a crucial role (21). For example, a study by Holmes et al. (17) reported that all investigated patients, who have formerly been suicidal, described the experiencing of vivid mental images of possible future suicide attempts. On the same note, suicidal ideation within this sample was related to the preoccupation with imaginations about suicide, as well as the perceived realness of imaginations. These findings are supported by another clinical study, which revealed that suicidal flash-forwards are present in suicidal patients, and that individuals who recently experienced suicidal ideation and flash-forwards thereof, reported a more severe ideation of suicide than those without the latter (22). Additionally, a recent study comparing participants diagnosed with borderline personality disorder (BPD) and major depressive disorder (MDD) found that all patients experienced intrusive images of suicide, however, BPD patients with comorbid PTSD symptoms experienced even more vivid images than participants solely suffering from MDD (23). Furthermore, the same study showed that mental images of suicide were linked to the amount of previous suicide attempts, aversive childhood experiences (i.e., traumata), as well as severity of suicidal ideation. When compared to healthy controls (HCs), individuals who previously experienced suicidal ideation or even an attempt, show a higher rate of negative prospective images (21), as well as a more vivid imagery starring suicide or the aftermath of death (17, 21, 24). Therefore, suicidal flash-forwards could possibly be termed as a marker for suicide risk (22).

Knowing that suicidal behavior is most likely to occur during a depressive episode (25), past research has also focused on prospective mental imagery and its effects within the context of affective disorders. Whereas it is widely known that people suffering from depression experience higher rates of negative intrusive memories (26, 27), newer findings showed that depressed patients also experience a higher level of prospective intrusive images, considering these were personally relevant (28). Both, the negative appraisal of the stated intrusions as well as mental imagery itself, have been suggested to be a maintenance factor of depression (29, 30). In contrast, people who were diagnosed with depression or displayed higher scores of dysphoria were also reporting lower vividness of positive prospective images (31, 32). Furthermore, prospective intrusive imagery has also been linked to higher scores of state anxiety and depression within a wide range of affective disorders (28). Moreover, a study by López-Pérez et al. (33) also showed that negative intrusive prospective imagery was a predictive factor for symptoms of depression and anxiety in a population of criminal offenders. Looking at the clinical picture of bipolar disorders, which are characterized by a fluctuation between depression and (hypo)mania, past research has indicated that not only people suffering thereof experience an elevated amount of intrusive prospective imagery but also people that are solely at risk for the disorder (34). Furthermore, bipolar disorder has been linked to an excess of prospective intrusive imagery, which is suggested to be a contributing factor to the above characterized mood instability—the hallmark feature of the disorder (24, 35, 36). This finding found further support by the research of McGill & Moulds (37), who indicated that hypomanic tendencies are associated with the daily use of mental imagery, as well as with the experience of prospective intrusive images.

Adding more scientific findings about prospective intrusive imagery within personality disorders, specifically within BPD, a recent study showed that 67% of all investigated BPD patients reported the presence of mental images of self-harm, however, only 9% of these images were retrospective, but 42% were prospective images of self-harm (38). Moreover, the same study revealed that these vivid imageries about self-harm were serving as precursors of the actual execution of self-harming behavior, suggesting that the assessment of intrusive prospective imagery could be an essential tool in the treatment of nonsuicidal self-harming behavior.

Lastly, research on prospective intrusive imagery has also found that patients with schizophrenia compared to HCs experience more intrusive prospective imagery which were correlated with anxiety symptoms and posttraumatic intrusions. However, no difference regarding the amount of nonaffective imagery was found between patients and controls (39). The aforementioned authors suggest that there might be underlying deficits within the disorder, such as weakened contextual integration, that could possibly leave the individual vulnerable and therefore prone to experiencing intrusive past and prospective mental imagery.

Taken together, these first findings on the impact of prospective mental images on the maintenance and exacerbation of mental disorders require the development of measures that allow the assessment of their occurrence. However, imagery processes, especially those related to future events, are not routinely assessed within the clinical setting. To date, there is only one self-report measurement within the English context to assess prospective intrusive imagery: The impact of future events scale (IFES; 11). The IFES is derived from the widely used impact of events scale (IES, 40, 41), measuring PTSD symptoms after a traumatic event in regards to three symptomatic clusters: re-experiencing, avoidance, and hyperarousal. The IFES itself consists of 24 items, whereas the first 22 items are identic to those of the IES, with the difference that the questions are phrased to assess symptoms related to imagining prospective events. Additionally, the authors of the IFES added two more positive items, as the original IFES was created to assess symptoms related to positive as well as negative prospective images of the future. Thus far, there has been no assessment of the factorial structure of the IFES and its subscales, however, the authors reported satisfactory alpha indices of.87 as well as a sufficient test-retest reliability of *r* = .73, *p* < .001 for the English IFES (34). To date, the IFES has been used in various clinical studies, and has proven to be applicable in a variety of contexts (see findings described above by 28, 33, 34, 37, 39). However, due to the lack of a scientific measurement of prospective intrusive imagery within the German context, the present study aims to provide a German version of the IFES and evaluate its translation and adaption as follows: (1) To translate the IFES into German with the most comparable fit to the original version, (2) to obtain an item analysis of the translated version, (3) to validate the German version in terms of assessment of convergent and divergent validity in relation to other measures, and (4) to report the measures sensitivity and specificity in identifying individuals with and without mental images based on adequate cutoff -scores.

## Material and Methods

### Participants

The total sample consisted out of 141 participants, divided into a clinical subsample consisting out of 68 inpatient and outpatient and an HC sample consisting out of 73 participants. The clinical subgroup (54% female) was diagnosed with either an affective or anxiety disorder according to ICD-10 (42). The mean age was *M* = 39.88 (*SD* = 13.38; *Range*: 19–67). 43% of the sample either had a high school or university degree. All patients were recruited from the Department of Psychiatry and Psychotherapy III at the University Clinic of Ulm. The clinical inpatient and outpatient received a questionnaire package after one of their weekly therapy sessions, completed them at home and returned the questionnaires the following week to their respective therapist. The HC sample consisted of 73 participants (92% female), all of whom where university students. The mean age was *M* = 24.73 years (*SD* = 3.45, *Range* = 20–43). German was the first language for the entire sample. All participants received a questionnaire package during one of their university classes and returned the latter to their respective professor upon completion. The study was approved by the Internal Review Board of the Medical Faculty of the University of Ulm. All participants provided written informed consent prior to participating in the study.

### Procedure

#### Translation, Psychometric Evaluation and Revision of the German IFES

According to the guidelines provided by Schmitt & Eid (43), the IFES was first translated into German by two independent bilingual speakers. In a second step, a team of clinical psychologist with expertise in mental imagery reviewed and optimized the items. Thereinafter, the systematic back-translation method was implemented by two other bilingual speakers in order to insure the linguistic equivalence of the questionnaires. Both, the original English version and the back-translated version showed high equivalence, and in a last step only minor changes were made to the German version of the questionnaire by the members of the research team. After gathering and entering the data, various item analysis—such as discriminatory power and item difficulty—were conducted, which shall serve as the basis for the subsequent adaption and revision of the German measurement.

### Measures

#### Measure Assessing Prospective Intrusive Imageries

*Impact of Future Events Scale* (IFES; 8) was used to assess intrusive prospective re-experiencing, hyperarousal, and avoidance. First, participants were asked to identify three future events that they have been thinking about during the past 7 days. Thereinafter, the participants were asked to choose one negative event from the created list about future events that they have been imagining and respond to each of the 24 items on a five-point Likert scale (0 = not at all; 4 = extremely) in regards to how much each of the statements was true for that specific negative event. Items included “Pictures about the future popped into my mind” or “I tried not to talk about the future.” This procedure is a slight variation of the original IFES, in which participants were asked to answer the 24 items indiscriminately regarding positive or negative future events. This method was chosen due to the high applicability and impact of negative events within a sample consisting of affective and mood disorders, for which the German IFES shall be designed. Deeprose et al. (34) reported satisfactory alpha indices of.87 as well as a sufficient test-retest reliability of *r* = .73, *p* < .001 for the English IFES version.

#### Measures Assessing Convergent Validity

*Beck Depression Inventory-Second Edition* (BDI-II; 44; German translation: 45). The BDI-II is a self-report measure, assessing symptoms of depression at hand of 21 questions, by asking how the respective person has been feeling during the past two weeks including today. The high internal consistency with Cronbach’s α of.90 (46) could be replicated by its German equivalent (47).

*Spontaneous Use of Imagery Scale* (SUIS; 48; German translation: ). The German version of the SUIS is an 18-item measurement, which assesses general imagery use in everyday situations on a five-point Likert-scale (1 = never appropriate, to 5 = completely appropriate). Although the German version of the SUIS demonstrated low internal consistency during the initial validation (Cronbach’s α = .66; 49), the questionnaire displayed good internal consistency in the present study (Chronbach’s α = .86).

*State-Trait Anxiety Inventory* (STAI; 50; German translation: 51). The State-Trait Anxiety Inventory–Trait Version was developed to assess trait anxiety. Within this questionnaire, participants rate 20 items on a four-point Likert scale (1 = almost never, to 4 = almost always) in regards to how they feel in general. The original English version achieved high internal consistencies of.90 and above (50). These results were identic within the German adaption of the measurement (51).

#### Measures Assessing Divergent Validity

*Questionnaire About Life Satisfaction* (FLZ; 52). The FLZ is a self-report measure, assessing 10 different areas of life satisfaction (Health, Job and Work, Financial Situation, Free Time, Marriage and Relationship, Relationship with Own Children, You as a Person, Sexuality, Friends/Acquaintances/Relatives, and Housing). Each of these 10 subscales consists of seven items, which are rated on a seven-point Likert scale (1 = very unsatisfied, 7 = very satisfied). The statistical sum score across all items creates an overall life-satisfaction score, whereas the areas “Job and Work,” “Relationship with Own Children,” and “Marriage and Relationship” are not included in the overall score, due to the low applicability and therefore response rate of some participants. The internal consistency as indicated by Cronbach’s α amongst all scales ranges between .82 and .95 (52).

*Life Orientation Test—Revised* (LOT-R; 53, 54; German translation: 55). The LOT-R is a 10-item measurement designed to assess optimism and pessimism, whereas three items assess optimism, three items pessimism, and four items serve as fillers. Each item is rated on a five-point Likert scale (0 = strongly disagree, 4 = strongly agree). For the German version of the LOT-R the internal consistency as indicated by Cronbach’s α was.75 for the overall scale (55).

### Data Analysis

Data analysis was performed using Excel (2015) and IBM SPSS Statistics 24 (56).

*Step 1: Item analysis and revision of the German version:* First, internal consistency was assessed using Cronbach’s α for the total sample and the clinical sample. Then, item analyses were carried out in terms of item difficulty and discriminatory power for the clinical sample. Based on these results, the revision of the German IFES was conducted: Accordingly, items with item difficulty < 1.0, or discriminatory power < .3 were excluded.

*Step 2: Validation of the revised German IFES:* After excluding items that did not meet the above stated criteria, convergent validity was established using Pearson correlation coefficients between the German IFES sum score and BDI-II, STAI-T, SUIS, whereas divergent validity was assessed based on Pearson correlations between the translated IFES and FLZ and LOT-R. The significance level was adjusted according to Bonferroni (5%/5 = 1,00%) to 1.00 to prevent alpha-inflation. Subsequently, Steiger’s Z test (57) was implemented to examine divergent validity, by means of assessing the relative strengths of the different correlation coefficients. Receiver operating characteristics (ROC) analysis was used to test sensitivity and specificity, since these parameters allow for making distinctions regarding individuals with and without a certain characteristic (58). Within the present study, ROC analysis was used to distinguish clinical populations suffering from affective or anxiety disorders from healthy individuals, by providing an optimal cutoff score by which individuals can be classified in either category with high accuracy.

## Results

### Item Analysis

Due to low discriminatory power (< .3), items 12, 13, and 24 have been excluded from further analysis. Furthermore, item 23 has been eliminated, due to low item difficulty (< 1.0) (see **Table 1**). Results for the German short version (20 items) of the IFES revealed an excellent internal consistency as indicated by Cronbach’s α = .93 within the clinical sample. The original version (24 items) achieved a Cronbach’s α = .91. Due to the previous findings, the following analysis shall be carried out with the 20-item short version of the measurement (indicated as IFES-S; Questionnaire can be found in the **Supplementary Material**).

**Table 1:** Item difficulty and discriminatory power of the IFES-S.

	Item difficulty	Discriminatory power
Item 1	2.4	0.553
Item 2	1.85	0.713
Item 3	2.12	0.474
Item 4	1.92	0.474
Item 5	2	0.35
Item 6	2.67	0.63
Item 7	2.83	0.685
Item 8	1.98	0.608
Item 9	2.29	0.806
Item 10	1.62	0.607
Item 11	2.37	0.428
Item 12	1.52	0.069
Item 13	1.04	0.22
Item 14	1.35	0.715
Item 15	1.63	0.65
Item 16	1.92	0.617
Item 17	1.48	0.597
Item 18	1.83	0.68
Item 19	1.69	0.538
Item 20	1.25	0.451
Item 21	1.63	0.587
Item 22	1.69	0.641
Item 23	0.6	0.308
Item 24	0.44	-0.07

### Convergent and Divergent Validity

Convergent and divergent validity of the German IFES-S was evaluated through data gathered within the clinical sample (N = 68). In order to prevent alpha-inflation, Bonferroni adjusted significance levels were imposed before conducting further calculations. Correlations between the IFES-S and the BDI-II (*r* = .62; *p* < .01), the IFES-S and the STAI-T (*r* = .68; *p* < .01), and the IFES-S and the SUIS (*r* = .45; *p* < .01) were highly significant (see **Table 2**). In order to test for divergent validity, the IFES-S was correlated with the FLZ and the LOT-R (pessimism subscale) respectively. Results indicated a moderate negative correlation between the IFES-S and both proclaimed measures (see **Table 2**). During further analysis, Steiger’s Z test (57) was performed to evaluate the relative strengths of the above described correlations between the IFES-S and the BDI-II, STAI-T, SUIS, FLZ, and LOT-R (pessimism subscale) respectively. The analyses revealed that the IFES-S total score was more strongly correlated with the STAI-T (Z = 5.89, p = .000), the BDI-II (Z = 5.55, p = .000), and the SUIS (Z = 5.35, p = .000) than with the FLZ. Moreover, the IFES-S total score was also more strongly correlated with the STAI-T (Z = 5.84, p = .000), the BDI-II (Z = 5.40, p = .000), and the SUIS (Z = 5.17, p = .000) than with the LOT_R.

**Table 2:** Zero-order correlations among IFES-S and BDI-II, STAI-T, SUIS, FLZ, and LOT-R.

Scores	Scores					
	IFES-S sum	BDI-II sum	STAI-T sum	SUIS sum	FLZ sum	LOT-R Pessimism sum
IFES-S sum	1.00	623**	.675**	448**	–.455**	–.418**
BDI-II sum		1.00	.875**	.250*	–.673**	–.633**
STAI-T sum			1.00	.353**	–.706**	–.627**
SUIS sum				1.00	–.176	–.141
FLZ sum					1.00	.608**
LOT-R Pessimism sum						1.00

IFES-S, Impact of Future Events Scale-Short; BDI-II, Beck Depression Inventory-II; STAI-T, State-Trait Anxiety Inventory-Trait Subscale; SUIS, Spontaneous Use of Imagery Scale; FLZ, Questionnaire about Life-Satisfaction; LOT-R, Life Orientation Test-Revised; * correlation is significant at the 0.05 level; ** correlation is significant at the 0.01 level.

### Sensitivity and Specificity

**Figure 1:**
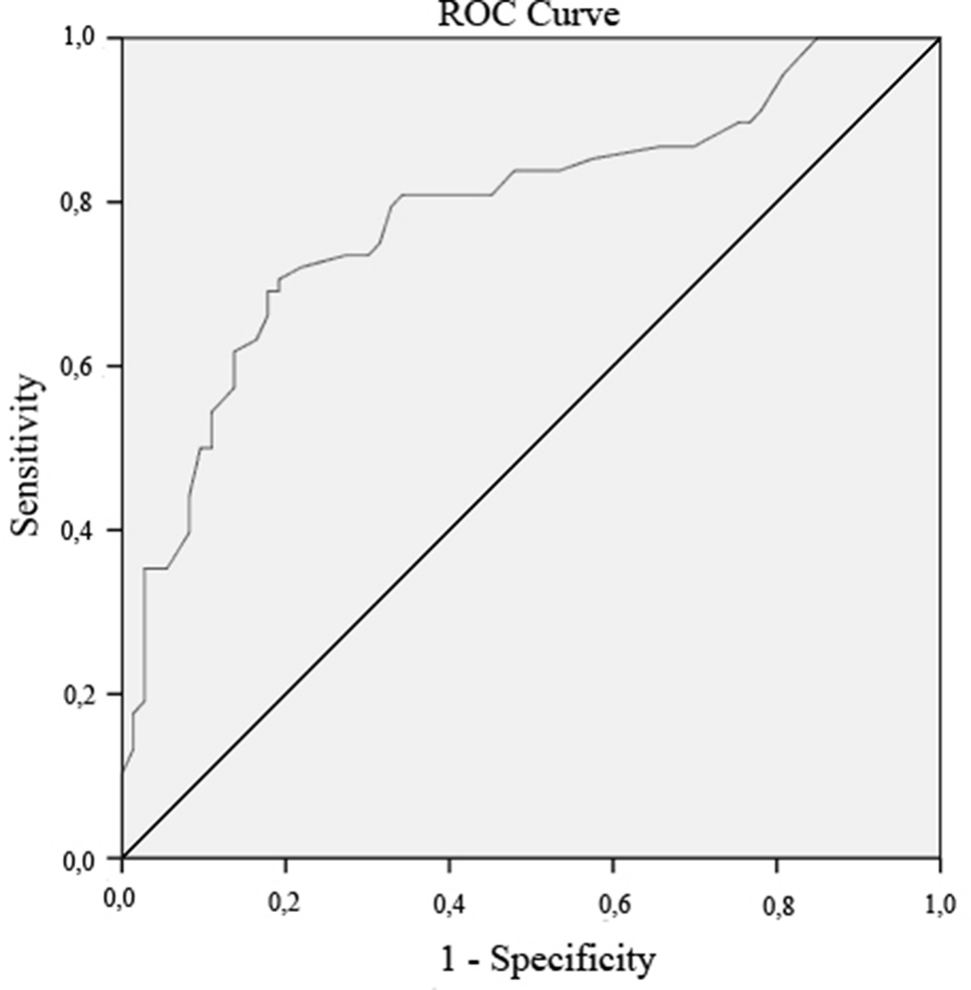
Receiver operating characteristic (ROC) curve. The ROC curve compares the clinical population with the healthy controls in regards to the German Impact of Future Events Scale-Short (IFES-S) score, and significantly discriminates between those two populations.

A ROC analysis was conducted to establish an optimal cutoff score, which is able to discriminate between clinical populations and HCs, as well as to evaluate the sensitivity and specificity of the German IFES-S (**Figure 1**). The area under the curve (AUC) was .79 (95% CI = .71–.86). An optimal balance between sensitivity and specificity was achieved at a cutoff score of 22.5 (rounded to 23 for clinical applicability), displaying a sensitivity of .81 and a specificity of .66. Therefore, 81% of all patients suffering from an affective or anxiety disorder were correctly classified, whereas 19% (1—sensitivity) were misclassified.

### Sample Characteristics

There was a significant age difference between the clinical sample (M = 39.88, SD = 13.38) and the HC sample (M = 24.73; SD = 3.45); t(65.72) = 8.53, p = .000 (see **Table 3**). Furthermore, the two samples showed significant differences in regards to sex *X^2^* (1, N = 141) = 35.44, *p = .*000, and level of education *X^2^* (3, N = 141) = 60.31, *p = .*000.

**Table 3:** Demographic Characteristics of the sample, as well as mean scores, standard deviations, and significance levels of the measures.

	Clinical Sample (*N* = 68)	Healthy Controls (*N* = 73)	Significance Level
Age (years)			
Mean	39.88	24.73	*T* = 8.53; *p* < .001**
SD	13.38	3.45	
Range	19-67	20-43	
Sex			*X^2^* = 35.44, *p* < .001**
Male	31 (46%)	6 (8%)	
Female	37 (54%)	67 (92%)	
Education			*X^2 ^*= 60.31, *p* < .001**
Secondary School	11 (16%)	0	
Middle School	28 (41%)	0	
High School	14 (21%)	22 (30%)	
University Degree	15 (22%)	51 (70%)	
IFES-S			
Mean	40.35	21.53	*T* = 7.19; *p* < .001**
SD	17.63	12.91	
BDI-II			
Mean	20.72	4.96	*T* = 10.75; *p* < .001**
SD	11.22	4.67	
STAI-T			
Mean	54.18	36.33	*T* = 10.37; *p* < .001**
SD	10.87	9.39	
SUIS			
Mean	52.13	54.99	*T* = -1.38; *p* = .17
SD	12.42	12.21	
FLZ			
Mean	173.07		
SD	37.67		
LOT-R Pessimism			
Mean	8.40		
SD	2.90		

SD, standard deviation; IFES-S, Impact of Future Events Scale-Short; BDI-II, Beck Depression Inventory-II; STAI-T, State-Trait Anxiety Inventory-Trait Subscale; SUIS, Spontaneous Use of Imagery Scale; FLZ, Questionnaire about Life-Satisfaction; LOT-R, Life Orientation Test-Revised; **, correlation is significant at the 0.01 level.

There was a significant difference between the clinical sample (M = 40.35, SD = 17.63) and the HC sample (M = 21.53, SD = 12.91) in regards to mean IFES-S scores *t*(122.24) = 7.19, *p* = .000, whereas the clinical sample achieved higher scores than the HC sample (see **Table 3**). Furthermore, the two groups showed significant differences with respect to their BDI-II scores, with the clinical sample (M = 20.72, SD = 11.22) displaying higher scores than the HC group (M = 4.96, SD = 4.67), t(88.20) = 10.75, p = .000. There was also a significant difference between STAI-T scores: The clinical sample (M = 54.18, SD = 12.42) displayed higher scores, than the HC sample (M = 36.33, SD = 9.39), *t*(132.55) = 10.37, *p* = .000. There was no difference between the groups in regards to SUIS scores *t*(137.93) = -1.38, *p* = .17.

## Discussion

The present study aimed to investigate the psychometric properties of the German IFES-S within a clinical sample of people suffering from either anxiety or affective disorders. Further goals were to evaluate convergent and divergent validities, as well as the measurement’s sensitivity and specificity, and lastly provide an optimal cutoff score to distinguish HCs from the clinical population.

After eliminating four items with either low discriminatory power or item difficulty, our results demonstrate good psychometric properties for the short version of the IFES (IFES-S). The study showed excellent internal consistencies, with a Cronbach’s α coefficient of.93 for the clinical sample. Our finding is consistent with the results of the original version (34), where a Cronbach’s α of.87 within a healthy sample for the English IFES questionnaire was reported. The here presented results also demonstrate good convergent validities, by significantly correlating with construct-related measures like SUIS, BDI-II, and STAI-T. These results are partially aligning with the findings by Deeprose & Holmes (11), who also found a significant positive correlation between the BDI-II and the English IFES. This finding is not surprising, considering the suggested maintenance factor of depressive symptoms depending on the negative appraisal of involuntary intrusive imagery (30). However, the same authors were unable to demonstrate a correlation between the IFES and the STAI-T. These differences might be due to the general nature of our study: Whereas Deeprose and Holmes (11) evaluated the IFES at hand of a HC sample, while categorizing this sample into a mild and a nondysphoric group, the present sample specifically consisted of people suffering from depression or anxiety. As the IFES is more likely to be applied to a clinical sample, we consider the here presented sample appropriate for validation purposes. Furthermore, Deeprose and Homes (11) validated the English IFES by having their participants fill out the questionnaire thinking about their imagery use in general during the past week (i.e., positive as well as negative prospective imagery). This procedure diverges from our protocol, where the German IFES-S shall be targeted at specifically measuring negative intrusive prospective imagery within a clinical group. Henceforth, the differences in regards to correlations between the English IFES, the German IFES-S, and the STAI-T might be due to the design of the study, the intended use of the IFES-S, and the targeted population. Moreover, correlations with measurements related to anxiety, depression, and imagery use (SUIS, BDI-II, STAI-T) were significantly higher than those with nonrelated measures (FLZ, LOT-R). This difference between related and unrelated measures can be interpreted as a supporting factor for the IFES-S’s satisfactory convergent and divergent validity. Third, sensitivity and specificity of the German IFES-S was evaluated and thereinafter an optimal cutoff score allowing to discriminate between patients suffering from depression or anxiety and a healthy population was determined. As expected, the German IFES-S provides an excellent discrimination between individuals suffering from affective or anxiety disorders and HCs, as can be seen by the highly significant AUC values. Furthermore, a cutoff score of 23 provided the ideal balance between specificity and sensitivity. Using this cutoff score, a sufficient correctly classified population can be identified. However, it is important to consider that the presented cutoff values are limited to a German population of people suffering from mood and anxiety disorders. Knowing that intrusive imagery also plays a crucial role within social phobia (6), or BPD (38), future studies could hence focus on adapting the IFES to the aforementioned psychiatric disorders. The here presented study further abstained from conducting a factorial analysis testing for the three-factorial structure as presented within the IES (40, 41), on which the original IFES (11) is based, due to the fact that the authors of the original IFES did not confirm this three-factorial structure. Furthermore, the results need to be interpreted with caution, since the assessed samples diverge in regards to age, sex, and education. Finally, future studies could extend onto the findings of this study by evaluating whether the IFES-S is sensitive, and therefore able to assess change within the treatment of mood and anxiety disorders.

Overall, the present findings demonstrate that the German IFES-S is a useful tool in evaluating negative intrusive prospective imageries within a clinical population of individuals suffering from affective or anxiety disorders. Our study further extends past findings on the English IFES (11, 34), confirming internal consistency coefficients, and providing additional information about psychometric properties, discriminant and convergent validities, as well as specificity and sensitivity of the adapted short version of the measurement. The German IFES-S in its final version could further be useful within the recently developing scientific field of prospective intrusive imagery, where to date, there has been no German measure to quantify intrusive prospective imagery. By allowing for the quantifiability of prospective intrusive imagery, this measurement will furthermore be helpful in evaluating the prevalence of those imageries within a healthy population as well as in various clinical disorders. Learning about the prevalence through research could subsequently support clinical practice by evaluating where intrusive prospective imageries are specifically relevant and eventually deleterious. However, it is not only by the means of research, that the IFES can be helpful within the clinical field: Within clinical practice it is fairly uncommon for patients to spontaneously report intrusive prospective imageries. By assessing those imageries through the IFES-S they become apparent to the therapist and therefore allow for the appropriate treatment, which might be especially important within the treatment of suicidality.

## Data Availability Statement

The datasets generated for this study are available on request to the corresponding author.

## Ethics Statement

The studies involving human participants were reviewed and approved by Internal Review Board of the Medical Faculty of the University of Ulm. The patients/participants provided their written informed consent to participate in this study.

## Author Contributions

JK: Study conception and design, analysis and interpretation of data, and manuscript writing and editing. CS: Study design, acquisition of data, and manuscript editing. AM: Study analysis of data and manuscript editing. BC: Study design and manuscript editing. ZS-V: Study conception and design, interpretation of data, manuscript editing, and critical revision.

## Conflict of Interest

The authors declare that the research was conducted in the absence of any commercial or financial relationships that could be construed as a potential conflict of interest.

## Supplementary Material

This material contains the full German IFES-S questionnaire.
