# Supplementary material for: The German Impact of Future Events Scale (IFES-S): Adaption and Validation for Clinical Samples
Source: Front Psychiatry. 2019 Nov 18;10:813. doi: 10.3389/fpsyt.2019.00813 (PMC6875826; doi:10.3389/fpsyt.2019.00813)
Supplement: Supplementary file 1 [file DataSheet_1.docx]

**Appendix I**

# **IFES-S**

**Anleitung**: Wenn man an Ereignisse in der Zukunft denkt, hat man oft nicht nur einen Gedanken wie „ich muss nächste Woche einen Vortrag halten“ im Kopf. Stattdessen stellt man sich bildhaft vor, wie die Situation ablaufen könnte. Man sieht quasi vor seinem inneren Auge, wie man vor den Zuhörern steht und es langsam ruhig im Raum wird, während man von allen erwartungsvoll beobachtet wird. Vielleicht stellt man sich auch vor, wie man anfängt zu schwitzen, dass man nicht mehr weiß, was man eigentlich sagen wollte, oder dass die Hände zu zittern beginnen. Oder man sieht beim Gedanken an das Mitarbeitergespräch am nächsten Tag den Chef schon vor sich sitzen und man kann fast hören, wie er die eigenen Fehler kritisiert, während man unruhig und angespannt vor ihm sitzt.

Bitte nennen Sie **drei zukünftige Ereignisse**, die Sie sich in den letzten 7 Tagen auf solche Weise bildhaft vorgestellt haben (z. B.: positive oder negative Lebensereignisse, davon mindestens ein negatives Ereignis). Bitte geben Sie für jedes Ereignis an, ob Ihre Vorstellung positiv oder negativ war, indem Sie die entsprechende Antwort einkreisen und geben Sie an, wie häufig Sie sich dieses Ereignis in den letzten 7 Tagen vorgestellt haben.

1. ____________________________________________________ (Positiv/Negativ)

Wie häufig haben Sie sich dieses Ereignis in den letzten 7 Tagen vorgestellt?

□ 1x □ 2-3x □ täglich □ mehrmals täglich

2. ____________________________________________________ (Positiv/Negativ)

Wie häufig haben Sie sich dieses Ereignis in den letzten 7 Tagen vorgestellt?

□ 1x □ 2-3x □ täglich □ mehrmals täglich

3. ____________________________________________________ (Positiv/Negativ)

Wie häufig haben Sie sich dieses Ereignis in den letzten 7 Tagen vorgestellt?

□ 1x □ 2-3x □ täglich □ mehrmals täglich

Im Folgenden sehen Sie eine Liste mit Aussagen, die von Leuten genannt werden, wenn sie sich Ereignisse in der Zukunft vorstellen. Bitte wählen Sie zunächst aus der oben erstellten Liste ein **negatives Ereignis** aus, welches Sie sich in den vergangenen 7 Tagen **bildhaft vorgestellt haben**. Lesen Sie anschließend jede Aussage und geben Sie an, wie häufig die jeweilige Aussage während der letzten 7 Tage zutraf, wenn Sie sich das ausgewählte zukünftige Ereignis vorstellten. Wenn Aussagen während dieses Zeitraums bei Ihnen nicht zutrafen, kreisen Sie bitte „überhaupt nicht“ ein.

Bitte kreisen Sie die Antwort ein, die am ehesten Ihrem Gefühl entsprach, wenn Sie sich während der letzten 7 Tage das zukünftige Lebensereignis vorstellten.

|  | Überhaupt nicht | Ein bisschen | Mäßig | Ziemlich | Sehr |
| --- | --- | --- | --- | --- | --- |
| 1. Ich dachte, dass meine Gedanken über dieses Ereignis mit Sicherheit eintreffen und wahr werden würden. | 0 | 1 | 2 | 3 | 4 |
| 1. Ich hatte Schwierigkeiten, nachts durchzuschlafen. | 0 | 1 | 2 | 3 | 4 |
| 1. Andere Dinge veranlassten mich, über das Ereignis nachzudenken. | 0 | 1 | 2 | 3 | 4 |
| 1. Ich fühlte mich reizbar und ärgerlich. | 0 | 1 | 2 | 3 | 4 |
| 1. Ich versuchte, mich nicht aufzuregen, wenn ich an das Ereignis dachte oder an es erinnert wurde. | 0 | 1 | 2 | 3 | 4 |
| 1. Auch ohne es zu beabsichtigen, musste ich an das Ereignis denken. | 0 | 1 | 2 | 3 | 4 |
| 1. Immer wenn mich etwas an das Ereignis erinnerte, wurden die dazugehörigen Gefühle wieder wachgerufen. | 0 | 1 | 2 | 3 | 4 |
| 1. Ich versuchte Erinnerungen an das Ereignis aus dem Weg zu gehen. | 0 | 1 | 2 | 3 | 4 |
| 1. Bilder des Ereignisses kamen mir plötzlich in den Sinn. | 0 | 1 | 2 | 3 | 4 |
| 1. Ich war leicht reizbar und schreckhaft. | 0 | 1 | 2 | 3 | 4 |
| 1. Ich versuchte, nicht an das Ereignis zu denken. | 0 | 1 | 2 | 3 | 4 |
| 1. Ich stellte fest, dass ich handelte oder fühlte, als ob es wirklich eingetreten wäre. | 0 | 1 | 2 | 3 | 4 |
|  | Überhaupt nicht | Ein bisschen | Mäßig | Ziemlich | Sehr |
| 1. Ich konnte nicht einschlafen. | 0 | 1 | 2 | 3 | 4 |
| 1. Es kam vor, dass die Gefühle, die mit dem Ereignis zusammenhingen, plötzlich für kurze Zeit viel heftiger wurden. | 0 | 1 | 2 | 3 | 4 |
| 1. Ich versuchte, Gedanken an das Ereignis aus meiner Erinnerung zu streichen. | 0 | 1 | 2 | 3 | 4 |
| 1. Es fiel mir schwer, mich zu konzentrieren. | 0 | 1 | 2 | 3 | 4 |
| 1. Gedanken an das Ereignis lösten bei mir körperliche Reaktionen aus, wie Schwitzen, schnelleres Atmen oder Herzrasen. | 0 | 1 | 2 | 3 | 4 |
| 1. Ich träumte von dem Ereignis. | 0 | 1 | 2 | 3 | 4 |
| 1. Ich fühlte mich wachsam und alarmiert. | 0 | 1 | 2 | 3 | 4 |
| 1. Ich versuchte, nicht über das Ereignis zu sprechen. | 0 | 1 | 2 | 3 | 4 |
